# Association of Household Food Insecurity With Dietary Intakes and Nutrition-Related Knowledge, Attitudes, and Practices Among School-Aged Children in Gaza Strip, Palestine

**DOI:** 10.3389/fnut.2022.890850

**Published:** 2022-06-29

**Authors:** Abdel Hamid El Bilbeisi, Ayoub Al-Jawaldeh, Ali Albelbeisi, Samer Abuzerr, Ibrahim Elmadfa, Lara Nasreddine

**Affiliations:** ^1^Department of Nutrition, School of Medicine and Health Sciences, University of Palestine, Gaza, Palestine; ^2^Regional Office for the Eastern Mediterranean (EMRO), World Health Organization (WHO), Cairo, Egypt; ^3^Health Research Unit, Palestinian Ministry of Health, Gaza, Palestine; ^4^Department of Social and Preventive Medicine, School of Public Health, University of Montreal, Montreal, QC, Canada; ^5^Department of Nutritional Sciences, Faculty of Life Sciences, University of Vienna, Vienna, Austria; ^6^Nutrition and Food Sciences Department, Faculty of Agriculture and Food Sciences, American University of Beirut, Beirut, Lebanon

**Keywords:** attitudes, dietary intakes, food insecurity, nutrition-related knowledge, practices, school-aged children

## Abstract

**Background:**

The present study aimed to determine the association of household food insecurity with dietary intakes and nutrition-related knowledge, attitudes, and practices (KAP) among school-aged children.

**Methods:**

This cross-sectional study was conducted among a representative sample of school-aged children. A total of 380 children and their parents were selected from all Gaza strip governorates, using a random sampling method. The demographic and socioeconomic characteristics; the Radimer/Cornell food security scale; two non-consecutive days of 24-h dietary recall; anthropometric measurements; and the Food and Agriculture Organization KAP-questionnaire (Module 3) were employed. Statistical analysis was performed using SPSS version 25.

**Results:**

About 71.6% of school-aged children were household food-insecure, while 28.4% were household food-secure. Significant associations were found between living area, educational level, household monthly income, weight for age and BMI for age z-scores, underweight, malnutrition status, intakes of protein, iron, vitamin D, and zinc among household food-secure, and household food-insecure. After adjustment for confounding variables, having nutrition-related adequate KAP were associated with lower odds of being food-insecure household [*OR* = 0.519, 95% (*CI* = 0.320–0.841)], [*OR* = 0.510, 95% *CI* = (0.315–0.827)], and [*OR* = 0.466, 95% *CI* = (0.285–0.763), *P* < 0.05 for all], respectively.

**Conclusions:**

Low socioeconomic status, low anthropometric indices, poor dietary intakes may be associated with a high level of food-insecurity; while having nutrition-related adequate KAP may be protective against food-insecurity among school-aged children.

## Introduction

Food insecurity is described as a scenario in which people do not always have access to enough, safe, and nutritious food to satisfy their dietary demands for an active and healthy life while also taking their food preferences into account (1, 2). Worldwide, the number of people affected by moderate or severe food insecurity will continue to increase in 2022. In addition, nearly one in three people worldwide did not have access to adequate food, with an increase of almost 320 million people in just 1 year (3). The high cost of healthy diets coupled with persistently high levels of income inequality put healthy diets out of reach for around three billion people, especially the poor, in every region of the world (4). Most of these people live in Asia (1.85 billion) and Africa (1.0 billion) (5).

Despite international and national efforts to eradicate severe poverty and enhance the global food supply, food insecurity continues to be a significant problem that affects people all over the world, especially those in low- and middle-income nations (6). According to recent research, food insecurity is concentrated mainly in conflict-affected countries experiencing political unrest, financial instability, and relocation (7, 8). Furthermore, the Palestinian situation was one in which war remained the primary cause of food insecurity, affecting the lives of two million of the population and exacerbated by high poverty and unemployment rates (9). Additionally, protracted conflict, economic stagnation and restricted trade and access to resources, coupled with high unemployment poverty rates, continue to pose serious challenges to the achievement of food security and improved nutrition (10). Nearly seven out of ten people in Gaza are impoverished, half of the workforce is jobless, and seven out of ten families are food insecure (11). Moreover, based on recent data, over 68 percent of households in the Gaza strip are food-insecure (12).

Food insecurity has the potential to be harmful to individuals of any age, but it can be especially devastating to school-aged children (13). School-aged children who experience food insecurity may suffer from low cognitive abilities poor academic performance, emotional, behavioral, mental, and physical consequences (14). Many of the previous studies show that food-insecure school-aged children are almost twice as likely to be in poor health when compared to food-secure children and are significantly more likely to be hospitalized (15, 16). On the other hand, nutrition-related knowledge, attitudes and practices (KAP) are necessary for dietary changes toward a healthier dietary pattern (17). For that reason, food and nutrition-related KAP is one of the key factors to achieving households food and nutritional security (18). In fact, earlier research has focused on young children (under the age of 5 years) (19, 20), with older children receiving less attention (16). Recent research suggests that school-aged children are just as exposed, if not more prone, to the negative repercussions of household food insecurity than their younger siblings (21, 22). Due to their reliance on the table food available in the home, school-aged children may be at an increased risk of health problems associated with food insecurity compared to their younger siblings, who may still be nutritionally protected by breastfeeding and other early infant feeding practices (23). Furthermore, school-aged children have more critical dietary requirements. However, compared to their younger children, moms may be less aware of their nutritional intakes or have less influence over their older children's food and beverage choices outside the house (24). Therefore, the present study was conducted to determine the association of household food insecurity with dietary intakes and nutrition-related KAP among school-aged children in the Gaza Strip, Palestine.

## Materials and Methods

### Study Design

This cross-sectional study was conducted in the year 2021 among a representative sample of school-aged children aged more than 5 to <18 years. A total of 380 school-aged children and their parents were selected from all Gaza strip governorates based on the population density, using a random sampling method.

### Eligibility Criteria

Households having at least one school-aged child (male or female), aged more than 5 to <18 years, and living with his/her mother in the same household, and mothers and fathers aged ≥18 years and having school-aged children were included in the present study. On the contrary, households without school-aged children, mothers and fathers with disabilities or chronic disease, and school-aged children with disabilities or chronic disease were excluded from the present study.

### Study Location

The current study was conducted in the households of the Gaza strip, Palestine. The estimated population of the Gaza strip is about 2,106,745 million. Gaza strip is divided into five governorates: North-Gaza, Gaza, Middle-Area, Khanyounis, and Rafah, with a population density of 19.3, 34.9, 14.4, 19.1, and 12.2%, respectively (25).

### Sample Size and Sampling

In the present study, the representative sample size was calculated using the following formula.


(1) $$\begin{array}{l} Sample\;size\;\left(n\right)\;=\;\frac{Z_{1-\alpha /2}^{2}\;P(1-P)}{d^{2}}\;=\\ \;\frac{{\left(1.96\right)}^{2}\left(0.50\right)\;(1-0.50)}{{\left(0.05\right)}^{2}}=380 \end{array}$$

Where, Z_1−α/2_ = Standard normal variate (Z value is 1.96 for a 95 percent confidence level); *p* = Response distribution (50%); and *d* = Margin of error (5%).

Accordingly, 380 school-aged children and their parents were recruited in this study, applying a random sampling method. The sample was distributed into the five governorates of the Gaza strip based on the population density as follows: 73 from North-Gaza, 133 from Gaza, 55 from Middle-Area, 73 from Khanyounis, and 46 from Rafah.

### Tools of the Study

#### Interview Questionnaire

To achieve the purpose of the study, an interview-based questionnaire was used; the data was collected from the school-aged children and their parents (mothers and fathers) by ten qualified data collectors who were given a full explanation and training by the researcher about the study. The data collectors went to the participants' homes, and the study participants did not go to any research center. The questionnaire contains items about the demographic and socioeconomic characteristics of the school-aged children; the 10-item Radimer/Cornell food security scale to assess the household food security status (26); two non-consecutive days of 24-h dietary recall for dietary intakes assessment; anthropometric measurements; and the Food and Agriculture Organization (FAO) of the United Nations questionnaire (Module 3: Diet of school-aged children) to assess the nutrition-related KAP of school-aged children (27). Before data collection, a pilot study was carried out on 30 participants to enable the researcher to examine the tools of the study. The questionnaire and data collection process were adjusted according to the result of the pilot study. In addition, during pilot study, test-retest reliability was undertaken, where the same tool was administered to the same participants twice and the results were compared for congruency.

#### Demographic and Socioeconomic Characteristics of School-Aged Children

An individual face-to-face interview was conducted with the school-aged children and their parents (mothers and fathers) to collect information about demographic and socioeconomic characteristics of school-aged children, including age (years), gender, governorate, the nature of the living area, educational level of the head of households (mothers or fathers), and monthly household income (NIS). The used categories of educational level and monthly household income (NIS) variables in the current study were similar to which mentioned in earlier studies in Gaza strip (28, 29).

#### Assessment of Households' Food Security Status

The 10-items Radimer/Cornell food security scale was used for determining the households' food security status. The scale is a valid and reliable tool for measuring household food insecurity in a culturally diverse setting (30), including Palestine (28, 29). An interview was conducted with the head of households (mothers or fathers) to collect data about the households' food security status. Then, the households were classified by food security status as follow: (1) Household food secure: Negative answers to all hunger and food insecurity items; (2) Household food insecurity: Positive answers (“sometimes true” or “often true”) to one or more hunger and food insecurity items.

#### Dietary Intakes Assessment of School-Aged Children

Two non-consecutive days of 24-h dietary recall were employed to determine the quantity of macro-and micronutrients consumed by the school-aged children. The school-aged children and their parents (mothers and fathers) were requested to recall all beverages and food consumed by the school-aged children in the past 24 h. In the present study, the data were collected on the first day of the two non-consecutive days of 24-h dietary recall by a face-to-face interview, and then the data collectors obtained each participant's phone number and called him/her later to obtain data about another different day. The portion sizes were estimated using a set of household measurements (i.e., plates, cups, glasses, and spoons). Dietary data from the 24-h dietary recall was processed by hand (office work) in order to calculate the net grams of foods consumed by school-aged children. This information was analyzed using the Nutritionist Pro Software version 7.1.0 (Axxya Systems, USA) (31) to determine energy (kcal) and nutrients intakes, including protein (g), carbohydrate (g), fat (g), iron (mg), vitamin A (μg), vitamin D (μg), calcium (mg), and zinc (mg). Additionally, the nutritional calculations were performed based on the USDA Food Composition Database.

### Anthropometric Measurements of School-Aged Children

#### Height and Weight

The height (cm) and weight (kg) of the school-aged children were measured following standard recommended procedures. A digital weighing scale (to the nearest 0.1 kg) (SECA, Germany) and a stadiometer (with the precision of 0.1 cm) were used (32). All measurements were taken twice, and the average of the two values was reported. The body mass index (BMI) was calculated by dividing weight in kilograms by the square of height in meters (33). Furthermore, the age, weight, height, and BMI of the children were translated into three indices: weight for age (WAZ), height for age (HAZ), and BMI for age (BMIZ), which were expressed in terms of z-scores using the WHO Anthro Software (Version 3, 2009) (34). The school-aged children were classified into moderate and severe underweight and moderate and severe stunting, which mean that WAZ and HAZ z-scores are < – 2 and < – 3, respectively (35). Then, the school-aged children were classified based on their malnutrition status into obesity, overweight, thinness, and severe thinness, which mean that BMIZ is> +2, > +1, < −2, and < −3 SD, respectively (36).

### Mid-upper Arm Circumference

Mid-upper Arm Circumference (MUAC) (cm) was recorded to the nearest 0.1 cm using the MUAC measuring tape. Investigators were measured the MUAC at the midpoint of the arm, where the measuring tape was snugged to the skin but not pressing soft tissues (37). All measurements were taken twice, and the average of the two values was reported.

### Assessment of Nutrition-Related KAP

The FAO of the United Nations questionnaire (Module 3: Diet of school-aged children) for assessing nutrition-related KAP of school-aged children was used to conduct high-quality surveys (17). Accordingly, we have used the same valid questionnaire distributed by the FAO in Palestine in 2017 (38). The questionnaire comprises predefined questions that capture information on critical KAP related to the diet of school-aged children. In the present study, the nutrition-related KAP consists of two questions related to nutrition-related knowledge, six questions related to nutrition-related attitudes, and 12 questions related to nutrition-related practices (39). Additionally, data regarding the nutrition-related KAP of school-aged children were collected from the head of households (mothers or fathers) using an interview-based questionnaire.

### Data Analysis

The Statistical Package for Social Science (SPSS) for Windows (version 25) was used for data analysis. Descriptive statistics were used to describe continuous and categorical variables. The chi-square test and fisher's exact test were used to determine the significant differences between categorical variables. The differences between mean were tested by independent samples *t*-test. Furthermore, crude and adjusted odds ratio (OR) and 95% confidence interval (CI) for the overall nutrition related-KAP of school-age children by household food-security status were calculated using binary logistic regression. A *P* < 0.05 was considered statistically significant.

## Results

Table 1 presents the demographic and socioeconomic characteristics of school-aged children by food security status. A total of 380 school-aged children (43.4 females and 56.6% males) were included in the current study. About 272 (71.6%) of the included households were food insecure, while only 108 (28.4%) were food secure. Nearly half of the food-insecure households, 131 (48.2%), were located at refugee camps. About 144 (52.9%) of the head of households (mothers or fathers) who belong to food-insecure households had a low education level. A large percentage of food-insecure households, 164 (60.3%), had monthly household income ≤2,000 New Israeli Shekel (NIS)—the local currency. There were statistically significant association differences between household food-secured and household food-insecured regarding the nature of the living area, educational level of school-aged children, and monthly household income (*P* = 0.036, 0.049, and 0.004, respectively).

**Table 1:** Demographic and socioeconomic characteristics of school-aged children by food security status.

**Variables**	**Household food-secure** **(*n* = 108)**	**Household food-insecure** **(*n* = 272)**	***P*-value**
**Age (years)**
Mean ± SD	10.85 ± 3.57	10.82 ± 3.58	0.952^a^
**Gender**
Males	60.0 (55.6)	155 (57.0)	0.800^b^
Females	48.0 (44.4)	117 (43.0)	
**Governorate**
North Gaza	15.0 (13.9)	58.0 (21.3)	0.063^b^
Gaza	35.0 (32.4)	97.0 (35.7)	
Middle Area	22.0 (20.3)	32.0 (11.8)	
Khanyounis	18.0 (16.7)	55.0 (20.2)	
Rafah	18.0 (16.7)	30.0 (11.0)	
**Living area**
City	39.0 (36.2)	103 (37.9)	0.036^b^^*^
Village	9.0 (8.3)	38.0 (14.0)	
Camp	60.0 (55.5)	131.0 (48.1)	
**Educational level of the head of households (mothers or fathers)**			
Low education	42.0 (38.8)	144 (52.9)	0.049^b^^*^
High education	66.0 (61.2)	128 (47.1)	
**Household monthly income (NIS)**			
≤ 2,000	48.0 (44.4)	164 (60.3)	0.004^b^^*^
> 2,000	60.0 (55.6)	108 (39.7)	

a *Independent Samples t-test*.

b *Chi Square Test*.

^*^Difference is significant at the 0.05 level (two tailed).

*NIS, New Israeli Shekel*.

*Difference is significant at the 0.05 level (two tailed)*.

In addition, Table 2 shows the anthropometric parameters and status of undernutrition among school-aged children by food security status. The mean weight (kg) of school-aged children from household food secure and household food insecure were 39.98 ± 17.01 and 33.40 ± 16.24, respectively. The mean WAZ of school-aged children from household food secure and household food insecure were 0.75 ± 1.05 and −0.37 ± 1.20, respectively. The mean BMIZ of school-aged children from household food secure and household food insecure were 1.94 ± 0.57 ± 1.05 and −0.18 ± 1.09, respectively. Only 10.0 (9.3%) school-aged children from food-secure households experienced moderate underweight (weight for age), compared to 227 (83.5%) children from food-insecure households. Moreover, three children (2.7%) from food-secure households experienced severe underweight (weight for age), compared to 20.0 (7.3%) children from food-insecure households. Five children belonging to food-secure households experienced thinness, whereas 98 children belonging to food-insecure households experienced thinness. Besides, none of the children belonging to food-secure households experienced severe thinness, whereas ten children from food-insecure households experienced severe thinness. Furthermore, the results show significant association differences between household food security and household food insecurity concerning weight (kg), WAZ, BMIZ, underweight (weight for age), and malnutrition status (*P* < 0.05 for all).

**Table 2:** Prevalence of undernutrition and anthropometric parameters of school-aged children by food security status.

**Measurements**	**Household food-secure** **(*n* = 108)**	**Household food-insecure** **(*n* = 272)**	***P*-value**
**Weight (kg)**
Mean ± SD	39.98 ± 17.01	33.40 ± 16.24	0.041^a^^*^
**Height (cm)**
Mean ± SD	137.66 ± 19.04	136.76 ± 20.58	0.697^a^
**MUAC (cm)**
Mean ± SD	22.52 ± 5.86	21.57 ± 4.78	0.102^a^
**WAZ (z-score)**
Mean ± SD	0.75 ± 1.05	−0.37 ± 1.20	**<** 0.001^a^^*^
**HAZ (z-score)**
Mean ± SD	−0.74 ± 1.26	−0.79 ± 1.51	0.760^a^
**BMIZ (z-score)**
Mean ± SD	1.94 ± 0.57	−0.18 ± 1.09	**<** 0.001^a^^*^
**Underweight (weight for age)**			
Normal	95.0 (88.0)	25.0 (9.2)	0.001^b^^*^
Moderate	10.0 (9.3)	227 (83.5)	
Severe	3.0 (2.7)	20.0 (7.3)	
**Stunting**
Normal	91.0 (84.2)	214 (78.7)	0.390^b^
Moderate	11.0 (10.2)	38.0 (14.0)	
Severe	6.0 (5.6)	20.0 (7.3)	
**Malnutrition status**
Obesity	0.0 (0.0)	0.0 (0.0)	**<** 0.001^c^^*^
Overweight	9.0 (8.3)	0.0 (0.0)	
Normal nutritional status	94.0 (87.0)	164 (60.3)	
Thinness	5.0 (4.7)	98.0 (36.0)	
Severe thinness	0.0 (0.0)	10.0 (3.7)	

*MUAC, Mid upper arm circumference; WA, weight for age z-score; HAZ, Height for age z-score; BMIZ, Body Mass Index for age z-score*.

*Moderate and severe underweight, and moderate and severe stunting mean that weight for age, and height for age z-scores are < −2 and < −3, respectively (34)*.

*Obesity, overweight, thinness, and severe thinness mean that BMIZ is > +2, > +1, < −2, and < −3 SD, respectively (35)*.

*^a^Independent Samples t-test*.

*^b^Chi square test*.

*^c^Fisher's Exact Test*.

^*^Difference is significant at the 0.05 level (two tailed).

*Difference is significant at the 0.05 level (two tailed)*.

On the other hand, Table 3 shows energy, macro and micronutrients intakes of school-aged children by food security status. The results show that the mean levels of protein (gram) intake among school-aged children from household food secure and household food-insecure were 53.64 ± 22.83 and 40.54 ± 24.57 g, respectively. The mean levels of iron (mg) intake among school-aged children from household food secure and household food-insecure were 7.21 ± 2.96 and 6.12 ± 3.14, respectively. The mean levels of vitamin D (μg) intakes among school-aged children from household food secure, and household food-insecure were 11.24 ± 5.44 and 10.18 ± 5.57, respectively. The mean levels of zinc intakes (mg) among school-aged children from household food secure, and household food-insecure were 5.79 ± 2.70 and 4.51 ± 2.38, respectively. The association differences found between household food security and household food insecurity regarding intakes of protein, iron, vitamin D, and zinc were significant (*P* < 0.05 for all).

**Table 3:** Energy, macro and micronutrients intakes of school-aged children by food security status.

**Variables**	**Household food-secure** **(*n* = 108)**	**Household food-insecure** **(*n* = 272)**	***P*-value^a^**
**Energy (kcal)**
Mean ± SD	1,982 ± 791	1,948 ± 763	0.082
**Protein (gram)**
Mean ± SD	53.64 ± 22.83	40.54 ± 24.57	0.047*
**Carbohydrate (gram)**
Mean ± SD	361.33 ± 170.14	385.63 ± 175.91	0.774
**Fat (gram)**
Mean ± SD	76.73 ± 30.64	65.27 ± 32.54	0.068
**Iron (mg)**
Mean ± SD	7.21 ± 2.96	6.12 ± 3.14	0.036*
**Vitamin A RAE (microgram)**
Mean ± SD	542.75 ± 245.04	512.51 ± 349.51	0.052
**Vitamin D (microgram)**
Mean ± SD	11.24 ± 5.44	10.18 ± 5.57	0.032*
**Calcium (mg)**
Mean ± SD	1,045.02 ± 597.09	1,002.25 ± 599.41	0.063
**Zinc (mg)**
Mean ± SD	5.79 ± 2.70	4.51 ± 2.38	0.033*

a *Statistical testing using Independent samples t-test*.

^*^Difference is significant at the 0.05 level (two tailed).

*Difference is significant at the 0.05 level (two tailed)*.

**Figure 1:**
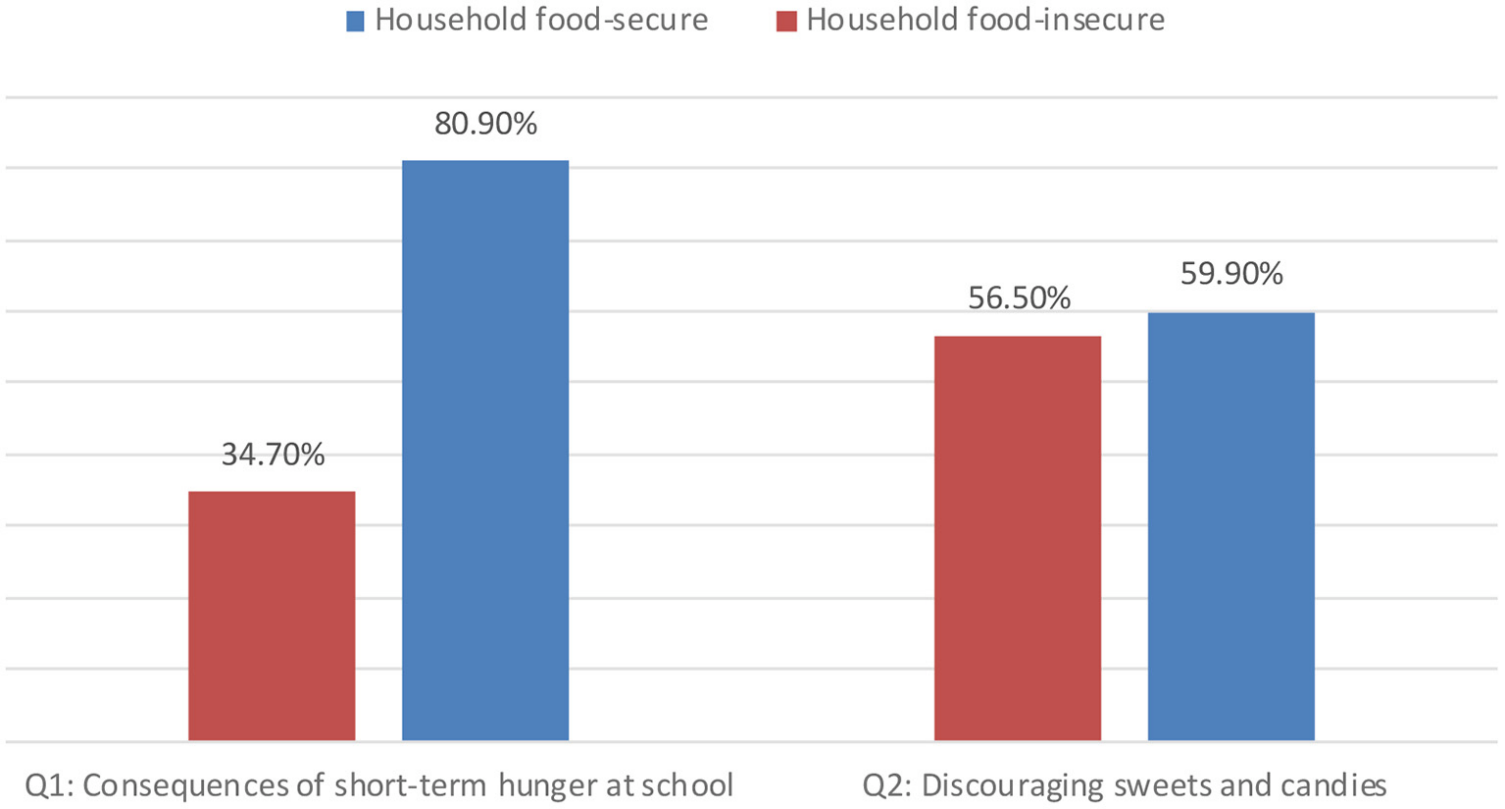
Nutrition-related adequate knowledge among household food-secure and household food-insecure.

Furthermore, Figure 1 shows the difference between household food-secure and household food-insecure in relation to nutrition-related adequate knowledge. About 59.9 and 56.5% of food-secure households and food-insecure households had adequate knowledge about the importance of discouraging children intake of sweets and candies. The majority of food-secure households (80.9%) had adequate nutrition-related knowledge about the consequences of short-term hunger at school, whereas only 34.7% of food-insecure households had that knowledge.

**Figure 2:**
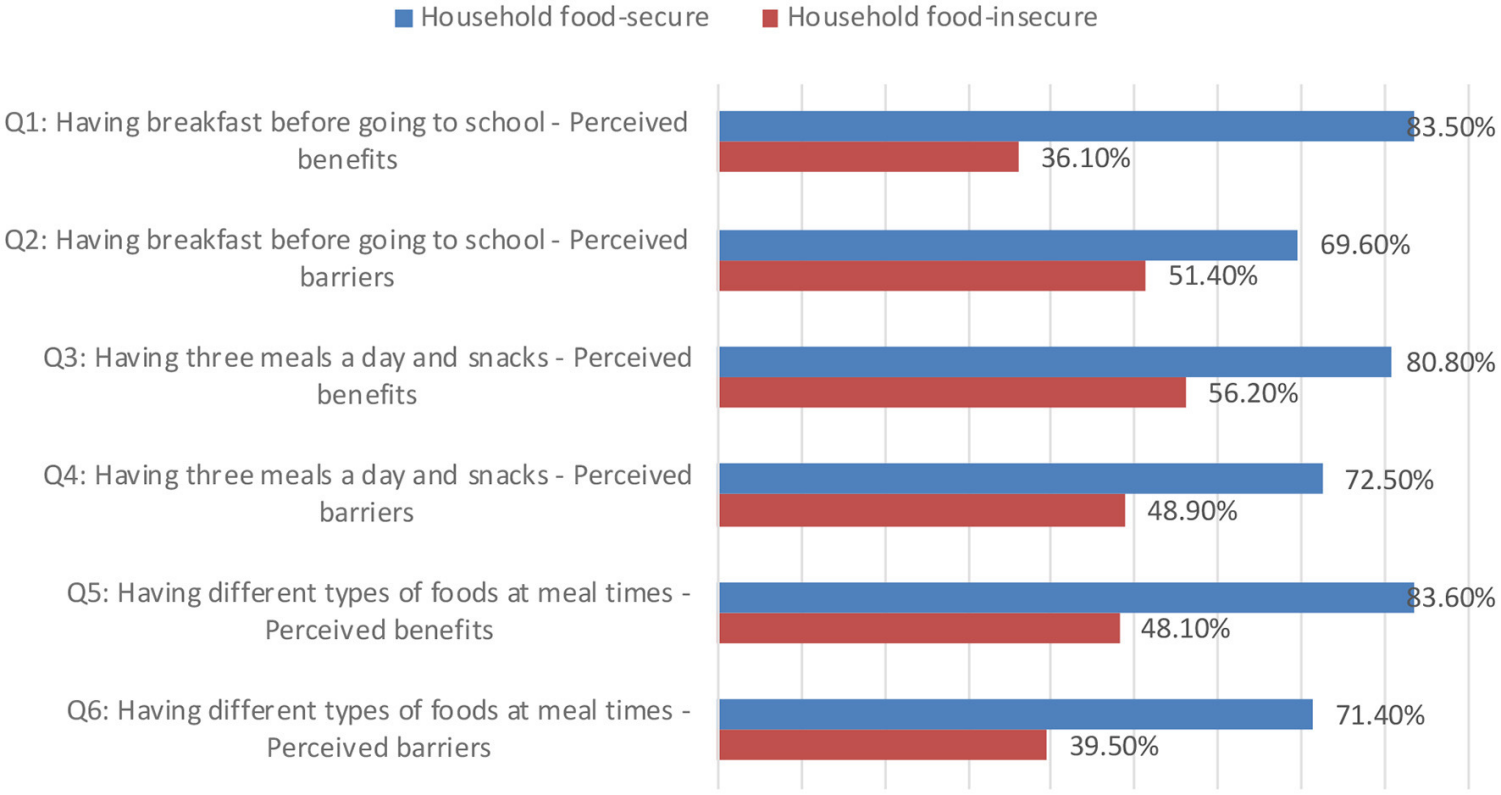
Nutrition-related positive attitudes among household food-secure and household food-insecure.

Moreover, Figure 2 shows the difference between household food-secure and household food-insecure concerning nutrition-related positive attitudes. The highest level of positive attitudes (83.6%) among household food security was related to having different types of foods at meal times-perceived benefit. The lowest level of positive attitudes (69.6%) was concerning having breakfast before going to school-perceived barriers. Moreover, the highest level of positive attitudes (56.2%) among household food insecure was related to having three meals a day and snacks-perceived benefits, while the lowest level of positive attitudes (36.1%) was concerning having breakfast before going to school-perceived benefits.

**Figure 3:**
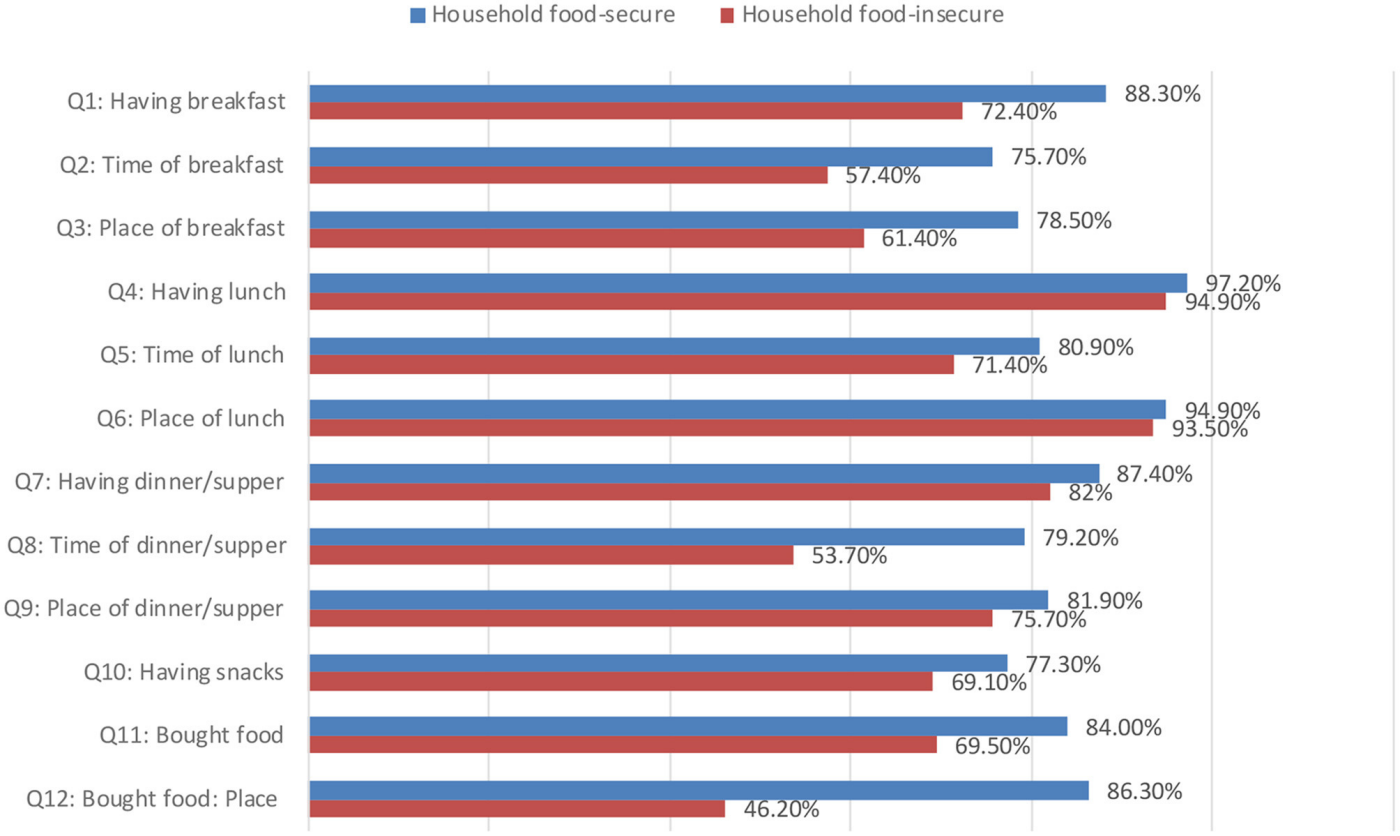
Nutrition-related good practices among household food-secure and household food-insecure.

Additionally, Figure 3 shows the difference between household food-secure and household food-insecure concerning nutrition-related good practices. The highest level of good practices (97.2%) among food-secure households was for the item related to (having lunch), and the lowest level of good practices (75.7%) concerned (time of breakfast). Furthermore, the highest level of good practices among the food insecure household (94.9%) was for the item related to (having lunch), and the lowest level of good practices (46.2%) was about bought food places.

Finally, Table 4 shows the crude and adjusted odds ratio (OR) and 95% confidence interval (CI) for the overall nutrition related-KAP of school-age children by household food security status. The results revealed that 70.4, 76.9, and 84.3% of food secure households had nutrition-related adequate knowledge, positive attitudes, and good practices, respectively; while for the food-insecure households, the results for these criteria were 45.6, 46.7 and 70.6%, respectively. After adjustment for confounding variables, having nutrition-related adequate knowledge, positive attitudes, and good practices were associated with lower odds of being food insecure household, compared to those with inadequate knowledge, negative attitudes and bad practices [*OR* = 0.519, 95% *CI* = (0.320–0.841), *P* < 0.05], [*OR* = 0.510, 95% *CI* = (0.315–0.827), *P* < 0.05], and [*OR* = 0.466, 95% *CI* = (0.285–0.763), *P* < 0.05], respectively.

**Table 4:** Crude and adjusted odds ratio and 95% confidence interval for the overall nutrition related-KAP of school-age children by household food-security status (*n* = 380).

**Food security status**	**Household food secure**		**Statistical tests**			
	**Yes *n* (%)** **108 (100)**	**No *n* (%)** **272 (100)**	
			**Crude OR (95% CI)**	***P*-value^a^**	**Adjusted OR (95% CI)^b^**	***P*-value^a^**
**Have adequate knowledge**						
No	32.0 (29.6)	148 (54.4)	Ref	–	–	0.008*
Yes	76.0 (70.4)	124 (45.6)	0.803 (0.511–1.261)	0.340	0.519 (0.320–0.841)	
**Have positive attitudes**						
No	25.0 (23.1)	145 (53.3)	Ref	–	–	0.006*
Yes	83.0 (76.9)	127 (46.7)	1.275 (0.799–2.034)	0.308	0.510 (0.315–0.827)	
**Have good practices**						
No	17.0 (15.7)	80 (29.4)	Ref	–	–	0.002*
Yes	91.0 (84.3)	192 (70.6)	0.996 (0.794–1.249)	0.971	0.466 (0.285–0.763)	

a *Statistical testing using binary logistic regression*.

b *Adjusted for the living area, educational level of school-aged children, and monthly household income (NIS)*.

*^ref^Reference*.

^*^Difference is significant at the 0.05 level.

*Difference is significant at the 0.05 level (two tailed)*.

## Discussion

To the best of our knowledge, this study was the first to evaluate the association of household food insecurity with dietary intakes and nutrition-related KAP among school-aged children in the Gaza Strip, Palestine. The findings of the present study may assist in establishing intervention programs aiming to improve the nutritional status of school-aged children experiencing food insecurity. More than two-thirds of our sample belonged to food-insecure households. Based on the recent data, over 68% of households in the Gaza strip are food-insecure (12). However, the results of our study are in line with the published data on some developing countries, such as Nepal (69%) (40) and Colombia (76%) (41). In addition, according to Elsahoryi et al. (42), 59.3% of Jordanian families were food insecure. The current analysis discovered a higher rate of food insecurity than the Food and Agriculture Organization (68.6%) (12). Food insecurity was prevalent in our study, which could be due to the Gaza Strip's extended war, economic stagnation, and limited trade and access to resources, as well as high unemployment and poverty rates (43).

In the present study, households of school-aged children with food insecurity had significantly lower monthly incomes than that of secure food households. In addition, food-insecure households were significantly associated with being located in refugee camps and low parental education levels of children. Previous research has consistently found that the poor socioeconomic status of families is connected with food insecurity (44, 45). Recent research found that lower-income households had a higher frequency of food insecurity (43%) than higher-income households; the study also found that families with parents with a low educational level had a considerably higher incidence of food insecurity (46). Another study found that children from low-income families had greater levels of household food insecurity, had more crowded households, and had moms who were less educated (47). Economic status can affect household food security status and expenditure for food through its effect on food accessibility. The high levels of education of parents were shown to be strongly related to food security in Saudi Arabia; higher education offers prospects for better employment and wages; therefore, parents' education is important (48).

In this study, body weight (kg), WAZ, BMIZ, underweight (weight for age), and malnutrition status were found to be linked with food security status where the other measured anthropometric were not. In addition, the results demonstrated that school-aged children from food-insecure households have a low mean of weight (kg), a high risk of moderate and severely underweight, and a high risk of thinness and severe thinness. Earlier studies have also examined these associations, and outcomes were conflicting. For instance, a study conducted among children in the United States and Saudi Arabia investigated the association of food security with weight status, and no association was observed (48, 49). Similarly, a study conducted in Bogota, Colombia, reported that children from food-insecure households were three times as likely to be underweight, while stunting was not associated with food insecurity of the household (41). Other studies showed significant associations between food insecurity and childhood obesity (50) and stunted growth (51). These controversial findings can be explained by the different methods for food security assessment, age group differences, and the reference for nutritional status assessment.

In this study, school-aged children protein (g), iron (mg), vitamin D (μg), and zinc (mg) intakes were significantly lower in food-insecure households than in food-secure households. Household access to food depends on the purchasing power of their income. When the income level is low, households resort to a number of coping strategies such as reducing the size of the meal, reducing the number of meals eaten, and eating low quality or cheap foods to gain access to food and protect food security levels (52, 53). Thus, restrictions in food intake, if repeated over a long time, may explain the lower nutritional status indices and lower nutrient intakes observed in the children living in food-insecure households.

On the other hand, a key finding in our study was that 70.4, 76.9, and 84.3% of food secure households had nutrition-related adequate knowledge, positive attitudes, and good practices, respectively; while for food-insecure households the values for these criteria were 45.6, 46.7 and 70.6%, respectively. Adequate nutrition-related KAP may be protective against food insecurity among school-aged children in the Gaza Strip, Palestine. Food insecurity is a significant nutritional issue worldwide and is commonly found in low- and middle- income countries like in Palestine. In addition, having physical and economic access to food on their own are not sufficient to ensure that people are food secure and well nourished. It is essential that people understand what constitutes a healthy diet; in particular, what nutrition-related health issues affect their communities, and how to address these through food-based approaches, and know how to make the best use of their resources. They should also have positive attitudes toward nutrition, diet, foods and closely related hygiene and health issues to be able to perform optimal dietary and feeding practices that ensure their nutritional wellbeing and that of their families. Finally, we found a positive association between parents' high level of education, and the high percentage of nutrition-related adequate knowledge, positive attitudes, and good practices with food security status of a household; may be this was possible because increased parents' education level could increase their knowledge about, attitude toward, and practice of healthy nutrition for the household members and specially for meeting the nutritional needs of the school-aged children.

### Strengths and Limitations

The main strength of our study was no indication of selection bias in this study, which included a representative sample of school-aged children. A valid and reliable tool (Radimer/Cornell food security scale) was used for determining the household food security status. Two non-consecutive days of 24-h dietary recall were employed to determine the quantity of macro-and micronutrients consumed. A digital weighing scale (SECA, Germany) and stadiometer were used as measurement equipment for anthropometric indices. Furthermore, the survey included a standardized nutrition-related KAP questionnaire that was suggested by the FAO. The main limitation of this study is its cross-sectional design; the causal relationship could not be determined, and it limits the generalizability of our results. In addition, the possibility of recall bias and misreporting by using of self-report retrospective data of 24-h dietary recall for the assessment of food consumption are other limitations.

## Conclusion

In conclusion, about 71.6% of school-aged children were in food-insecure households, and 28.4% were in food-secure households. Only 45.6, 46.7, and 70.6% of the food-insecure households had adequate nutrition-related knowledge, positive attitudes, and good practices, respectively. Additionally, low socioeconomic status, low anthropometric indices, poor dietary intakes may be associated with a high level of food insecurity, while having adequate nutrition-related KAP may be protective against food insecurity among school-aged children in the Gaza Strip, Palestine. Policy makers should continue to focus attention and investments in the most appropriate combinations of interventions to mitigate food insecurity level among school-aged children in the Gaza strip.

## Data Availability Statement

The raw data supporting the conclusions of this article will be made available by the authors, without undue reservation.

## Ethics Statement

The study protocol was approved by the Palestinian Health Research Council (Helsinki Ethical Committee of Research Number: PHRC/HC/961/21), University of Palestine Ethical Committee of Research, the Palestinian Ministry of Health, and Ministry of Interior. Further, written informed consent was obtained from each participant or their parents. Written informed consent to participate in this study was provided by the participants' legal guardian/next of kin.

## Author Contributions

AE collected, analyzed, and interpreted the data and wrote the first draft of the manuscript. AE, AA-J, AA, SA, IE, and LN significantly contributed in the study design and the critical review of the manuscript. AE and AA-J remarkably contributed to the analysis and interpretation of data and the critical review of the manuscript. All authors contributed to the article and approved the submitted version.

## Author Disclaimer

The authors alone are responsible for the views expressed in this article and they do not necessarily represent the views, decisions or policies of the WHO or the other institutions with which the authors are affiliated.

## Conflict of Interest

The authors declare that the research was conducted in the absence of any commercial or financial relationships that could be construed as a potential conflict of interest. The reviewer MS declared a shared affiliation with the author IE to the handling editor at the time of review.

## Publisher's Note

All claims expressed in this article are solely those of the authors and do not necessarily represent those of their affiliated organizations, or those of the publisher, the editors and the reviewers. Any product that may be evaluated in this article, or claim that may be made by its manufacturer, is not guaranteed or endorsed by the publisher.
