# Supplementary material for: Association of Household Food Insecurity With Dietary Intakes and Nutrition-Related Knowledge, Attitudes, and Practices Among School-Aged Children in Gaza Strip, Palestine
Source: Front Nutr. 2022 Jun 29;9:890850. doi: 10.3389/fnut.2022.890850 (PMC9277343; doi:10.3389/fnut.2022.890850)
Supplement: Supplementary file 1 [file Data_Sheet_1.PDF]

Radimer / Cornell Hunger and Food Insecurity Items

No.	Question	Not True	Sometimes True	Always True
	**A. Household level**			
**1.**	I worry that if the food or raw materials for cooking will run out before I could have more money to buy food.			
**2.**	Food or raw materials for cooking that I bought for my family at home is always run out fast and I do not have the money to buy food again.			
**3.**	I do not have enough food or raw materials to cook or prepare a family meal (for the tomb of the morning, noon or night) and I did not have enough money to buy food.			
**4.**	We eat the same thing for several days in a row because we only have a few different kinds of food on hand and do not have money to buy more.			
	**B. Individual level (Adult)**			
**5.**	I am often hungry but I do not eat because I do not have enough money to buy food.			
**6.**	I only eat a little of what I should eat because I do not have enough money to buy food.			
**7.**	I was not able to eat properly or eat to satiety because I do not have enough money to buy food.			
	**C. Individual level (Child)**			
**8.**	I am not able to provide a balanced meal to my children because I do not have enough money to provide food.			
**9.**	My children do not eat enough or always lack of food because I am not able to buy enough food.			
**10.**	I know sometimes my children are hungry, but I cannot do anything because I am not able to buy food in excess of what I always buy			

Socio-demographic characteristics for school-aged children (5-18 years old)		
**1.**	**Child name**	………………………………………
**2.**	**Sex**	☐ Male ☐ Female
**3.**	**Age (years)**	………………………………………
**4.**	**Governorate**	☐ North Gaza ☐ Gaza ☐ Middle Area ☐ Khan Yunis ☐Rafah
**5.**	**Where do you live?**	☐ City ☐ Village ☐ Refugees camp
**6.**	**Educational level of the head of households**	☐ Illiterate ☐ Primary ☐ Pr ☐ Secondary ☐ University
**7.**	**Family income (NIS)**	………………………………………

Anthropometric measures		
**1.**	Height (cm)	………………………………………
**2.**	Weight (kg)	………………………………………
**3.**	Mid Upper Arm Circumference (cm)	………………………………………

KAP about: Diet of school-aged children (5-18 years old)
**Knowledge**
**1. What problems can children have if they do not eat before going to school؟**
☐ Children have short attention/have low concentration/cannot study well/do not do as well at school as they should<br>☐ Other ☐ Don't know
**2. Why is it so bad to eat too many sweets and candies؟**
☐ Because they can cause tooth decay ☐ Because they are not nutritious ☐ Because they interfere with appetite<br>☐ Other ☐ Don't know
**Attitudes**

3. **How good do you think it is to have breakfast before going to school؟**		
☐ Not good	☐ You’re not sure	☐ Good
**4. How difficult is it for you to have breakfast before going to school؟**		
☐ Not difficult	☐ So-so	☐ Difficult
**5. How good do you think it is to have three meals a day and snacks؟**		
☐ Not good	☐ You’re not sure	☐ Good
**6. How difficult is it for you to have three meals a day and snacks؟**		
☐ Not difficult	☐ So-so	☐ Difficult
**7. How good do you think it is to have different types of foods at meals؟**		
☐ Not good	☐ You’re not sure	☐ Good
**8. How difficult is it for you to have different types of foods at meals؟**		
☐ Not difficult	☐ So-so	☐ Difficult

**Practices**		
**9. Did you have breakfast before going to school؟**		
☐ Yes	☐ No	☐ Don’t know/no answer
**10. If Yes: At what time؟**		
☐ Between 6 a.m. and 9 a.m	☐ Between 9 a.m. and noon	☐ Between noon and 3 p.m
**11. Where؟**		
☐ Home	☐ School	☐ Elsewhere
**12. If the interview is being conducted before lunchtime, ask: Did you have lunch yesterday؟ If the interview is being conducted after lunchtime, ask: Did you have lunch today؟**		
☐ Yes	☐ No	☐ Don’t know/no answer
**13. If Yes: At what time؟**		
☐ Between 9 a.m. and noon	☐ Between noon and 3 p.m	☐ Between 3 p.m. and 6 p.m

**14. Who prepares your lunch؟**		
☐ Parents at home<br>☐ Lunch is bought with pocket money	☐ School cafeteria<br>☐ Other	
**15. Did you have dinner yesterday؟**		
☐ Yes	☐ No	☐ Don’t know/no answer
**16. If Yes: At what time؟**		
☐ Between 3 p.m. and 6 p.m	☐ Between 6 p.m. and 9 p.m	☐ Between 9 p.m. and midnight
**17. Where؟**		
☐ Home	☐ School	☐ Elsewhere
**18. Yesterday during the day and night, did you eat anything between the meals؟**		
☐ Yes	☐ No	☐ Don’t know/no answer
**19. Yesterday during the day and night, did you buy foods with your own money؟**		
☐ Yes	☐ No	☐ Don’t know/no answer
**20. Where did you buy those foods؟**		
☐ At school/cafeteria	☐ On the street (from street vendors)	☐ Other
